# Thermodynamics of Peptide-MHC Class II Interactions: Not all Complexes are Created Equal

**DOI:** 10.3389/fimmu.2013.00308

**Published:** 2013-10-01

**Authors:** Andrea Ferrante

**Affiliations:** ^1^Molecular Immunology, Institute of Arctic Biology, University of Alaska Fairbanks, Fairbanks, AK, USA

**Keywords:** antigen presentation, MHC class II, peptide binding, thermodynamics

## Abstract

The adaptive immune response begins when CD4+ T cells recognize antigenic peptides bound to class II molecules of the Major Histocompatibility Complex (MHCII). The interaction between peptides and MHCII has been historically interpreted as a rigid docking event. However, this model has been challenged by the evidence that conformational flexibility plays an important role in peptide-MHCII complex formation. Thermodynamic analysis of the binding reaction suggests a model of complexation in which the physical-chemical nature of the peptide determines the variability in flexibility of the substates in the peptide-MHC conformational ensemble. This review discusses our understanding of the correlation between thermodynamics of peptide binding and structural features of the resulting complex as well as their impact on HLA-DM activity and on our ability to predict MHCII-restricted epitopes.

## Epitope Selection as a Thermodynamics-Based Process

Upon immunization with a complex antigen, or challenge by a pathogen, an organism’s CD4+ T cells recognize antigen-derived peptides bound to MHC class II molecules (MHCII) exposed on the surface of antigen presenting cells (APCs). A critical feature of the elicited T cell response is the phenomenon of immunodominance (1). This refers to the evidence that, despite the diversity of the T cell repertoire, the co-expression of different MHCII alleles and the potential generation of a panoply of distinct peptides, a large proportion of the responding T cells tends to be specific for a few peptide determinants, known as immunodominant antigens. A relevant observation related to immunodominance is the evidence that complex antigens have many sequences that can bind to the MHC molecule and trigger a T cell response if exogenously administered as single peptides. These latter sequences, which can be recognized only after immunization with the peptide and not after processing of the full protein, are known as “cryptic.” Efforts have been made to understand the mechanisms behind these phenomena. Analysis of the antigen processing and presentation pathway indicates that elements of the epitope selection process are critical to the generation of the peptide repertoire presented to T cells (2). MHCII molecules are transported from the endoplasmic reticulum to the MHCII compartments (MIIC) as multimeric complexes with the chaperone protein invariant chain (Ii). Ii stabilizes the nascent MHCII and prevents binding of other endoplasmic reticulum-resident peptides. Upon arrival in the MIIC, the Ii molecule is cleaved by proteases, leaving a low-affinity peptide fragment named class II-associated invariant peptide (CLIP) in the MHCII binding groove. CLIP is then released to allow peptides derived by endosomal fragmentation of antigenic proteins to be selected for presentation. Evidence collected thus far in the analysis of immunodominance indicates that a significant correlation exists between affinity and kinetic stability of a peptide-MHCII dyad, its density on the surface of the priming APC and the selection of a responding T cell repertoire (1, 3, 4).

The multitude of peptides, which can be derived from a protein, includes a consistent number of low-affinity ligands for a given MHC allele, as well as peptides with intermediate affinity and few high-affinity ones. Thus, under an immunodominance-based model of T cell response, MHCII molecules must reach a thermodynamic equilibrium in which they are complexed with the highest affinity binders. Considering that the transit time of an MHCII through the MIIC is comparable to the dissociation rate of many low-intermediate affinity peptides, it is difficult to presuppose that the MHC-binder dyad is able to reach such equilibrium intrinsically. Rather, one would expect that the majority of MHC molecules is bound to low-affinity peptides that are in excess (including CLIP) as a consequence of a kinetic control of the selection process (5, 6). So what can possibly explain the evidence of immunodominance? One reasonable hypothesis would be the existence of mechanisms that can enhance the thermodynamic control of the peptide-binding process.

HLA-DM (DM) is a MIIC-resident MHCII-like molecule that plays an important role in MHCII-restricted epitope selection. In the presence of DM, CLIP removal from the newly synthesized MHCII is facilitated, and the presented peptide-MHCII repertoire is skewed in favor of kinetically stable, high-affinity peptide-MHCII complexes. Based on these well-known effects, DM could be considered the likeliest factor determining the thermodynamic equilibrium of the endosomal machinery. In consideration of the impact of binding energetics on the outcome of epitope selection, this article will review of the thermodynamics of peptide interaction with MHCII, its correlation to complex structure and how these relate to DM activity.

## Some Structural Background

MHCII binds peptides in a groove defined by a β-sheet floor and two parallel helical sides. The groove is characterized by hydrophobic pockets and by a network of H-bonds that can form between MHCII side chains and the peptide backbone (7). MHCII show extensive polymorphism predominantly restricted to the peptide-binding groove. The contacts observed in available MHCII structures have helped cement an interpretation of MHCII-peptide-binding specificity based on hydrophobic pocket structure and charge matching (8). This interpretation reflects the historical model for explaining specificity of ligand-receptor interaction, namely the docking mechanism, which relies on structural rigidity and complementarity of shape (and possibly charge). The energetic contribution of each interaction – hydrophobic or H-bonding – has been evaluated by mutating individually single anchor residues or individually destabilizing specific H-bonds (9–11). Attempts have been made to predict peptide affinity by adding the energetic contribution to binding of each single interaction expected to be formed between the peptide and the MHCII. Indeed, several epitope prediction algorithms have been engineered on the assumption that peptide binding is a rigid, enthalpy-based process (12–16), but their specificity and sensitivity are questionable (17–19).

Biochemical and biophysical analyses have indicated an enhanced conformational heterogeneity or flexibility of the protein in the absence of a peptide (20–23). Several studies indicated that MHCII molecules undergo a conformational transition during peptide binding or exchange from a heterogenic state to a more rigid and stable state (21, 24, 25). The latter group of reports suggests that the mechanistic details of the peptide loading process are more complicated than those provided by a simple enthalpy-based docking model, and further analysis has confirmed this (26, 27). The ability to correlate MHCII and peptide structural rearrangement occurring during the interaction with the energetics of the binding process would provide important insights into the mechanisms controlling epitope selection. Whereas the literature concerning the thermodynamic properties of peptide-MHCII formation and their structural correlates is not extensive, most published observations indicate that complex formation can be interpreted as an instance of protein folding, and they will be reviewed in what follows.

## Cooperative Effects Impact Peptide Binding and Complex Kinetic Stability

The correlation between structural aspects of a peptide-MHCII complex and its thermodynamic features has been analyzed initially by thermal denaturation studies. Thermal stability of murine (28, 29) and human (20, 21, 30) MHCII, both empty and bound to different peptides at varied pH conditions, indicated that empty class II molecules have a different structure in terms of helicity as compared to bound structures. Moreover, binding of peptides with different length or sequence may result in complexes with different structure, and the derived thermodynamic parameters indicate that affinity of the peptide impacts the folding and unfolding of the complex.

The question of peptide binding as an instance of protein folding has been approached in recent times by probing cooperativity (31). Cooperativity in a multipoint ligand-receptor binding event is measured when one interaction is affected by other interactions. A general strategy to probe the occurrence of such a phenomenon is the mutant cycle approach (32). Mutant cycle analysis has been performed extensively in other systems to characterize transition states of folding and folding intermediates. This method consists of introducing multiple substitutions in the sequence of the reactants and assessing their binding parameters. If the effect on the binding free energy of the double (or multiple) mutation is not equal to the sum of effects of the single mutations, then the residues are coupled (cooperative). The first analysis of cooperativity with this strategy was performed on the I-Ad/HA (126–138) system (33, 34). The I-Ad MHCII protein binds peptides with high-affinity also in the absence of strong anchor, particularly relying on H-bonds. This first analysis indicated that the energetic contribution of a given anchor-pocket interaction is a function of the peptide sequence, suggesting that the binding energy is indeed distributed across the peptide, with a significant fraction sequestered in the N-terminal side of the complex. Whereas the H-bond network showed cooperativity, no detectable cooperativity between the anchor-pocket and H-bonding interactions was observed, even at a short distance, suggesting that for this allele, encapsulation of peptide side chains into MHCII pockets is independent and probably upstream of H-bond formation.

A similar strategy was applied to determine the extent of cooperativity the HLA-DR1 (DR1)/HA (306–319) system undergoes during complex formation or peptide release (35, 36). Several clusters of disruptive mutations have been applied to the peptide or the peptide and MHC up to a five-mutant construct, and they have evidenced cooperative effects in peptide binding to and release from MHCII. Due to the disruptive nature of the substitutions, cooperativity has been interpreted in terms of lack of folding (i.e., what is the effect of destabilizing a second interaction on the folding of the complex once a first interaction has been disrupted?). Cooperative effects impact peptide affinity or complex stability in an exponential fashion (35, 36). Differently than in the case of I-Ad, energetic coupling was observed between anchor/pocket interactions and H-bonds, when respectively located at opposite sides of the binding groove. A possible explanation for the different cooperativity in the two systems may be related to the evidence that the two alleles are differentially reliant on sequestration of peptide hydrophobic side chains into pockets for tight binding. This discrepancy, however, highlights the difficulty of finding a model of peptide-binding valid for all the class II alleles.

## Different Peptides Bind with Different Thermodynamic Strategies

Analysis of cooperativity has indicated that, prior to the exponential increase phase, an energy window exists, in which cooperativity (lack of folding) cannot be measured (35, 36). Within this window, indicated as compensatory region, peptides with different chemistry can bind with relatively comparable free energy decrease, though the relative enthalpic and entropic contributions differ significantly. This phenomenon, known as (isothermal) entropy-enthalpy compensation, has been observed in several flexible binding systems, in which a decrease (or increase) in the ability of forming binding interactions (enthalpy) can be counterbalanced by the increase (or decrease) of search of conformational space (37).

Following the spectroscopic interpretation of the interplay between cooperativity and entropy-enthalpy compensation (38), important insights have been gained into the correlation between peptide-MHCII complex structure and thermodynamics (37). As the interaction between peptides and MHCII molecules can be considered a ligand-receptor multipoint binding, each single interaction leads to a local modification of both structure and energetics. Such local modification can be thermodynamically described for each point system, which is defined by all the atoms within the interacting core whose radius is determined by the structural features of the same protein. While peptide and MHCII molecule interact, each point system will be affected by other point systems (cooperativity) to an extent proportional to the flexibility of the molecules and the distance between the two points. Since the protein and in particular some regions of the binding groove fluctuate, then the effect of flexibility on energetic coupling of multiple point interactions is correlated to the range and frequency of fluctuations, which change as new interactions are established. Fluctuating interactions are associated with a component energy and entropy. The enthalpic term of binding energy describes the stored energy in the protein due to conformational change associated with the binding, whereas fluctuations of the entire protein manifest themselves in the conformational entropy term. As new point systems are engaged in interactions, the enthalpic contribution to free energy of binding increases. The conformational change associated with one interaction would facilitate formation of a new cluster of interaction (positive cooperativity). The entropic contribution to binding decreases, however, since any new interaction also restrains conformational flexibility; therefore, the net gain in binding energy by cooperativity is minimal/null. For this reason high-affinity peptides cannot be constructed just by adding more and more sources of binding. Indeed it has been shown that the same high-affinity peptide binds better in the absence of an H-bond, as compared to when the same H-bond is present (37).

Importantly, it was found that the compensatory mechanism is not absolute, that is increasing in conformational flexibility consequent to lack of viable interactions between peptide and MHCII cannot overcome extremely poor enthalpic contributions. This phenomenon was evidenced by identifying a peptide affinity limit above which cooperativity (as exaggerated lack of folding) increased exponentially upon addition of disruptive mutation to the complex (37).

Thus, within the compensatory region, different peptides may form a complex with MHCII displaying similar IC50 in equilibrium-based competition binding assays, or comparable Kd in titration calorimetry, but with different enthalpic and entropic contributions. The diversity in thermodynamic strategy adopted by the system will reflect in the range and frequency of the fluctuations of each point system and the structure of the complex overall. The consequence is that the macrostate of a complex whose formation is highly enthalpic and whose conformational mobility is greatly restrained will be a collection of similar peptide-MHCII dyads where most if not all point systems is engaged in interactions with minimal liability at every point in time. On the contrary, complexes whose formation is correlated to a smaller entropic penalty are a collection of dyads whose greater conformation mobility is attempting to optimize the available interactions leading, at the same time, to structural variability. This is why postulating that binding of different peptides to a given MHCII results in “equal” complexes from thermodynamic and possibly structural standpoints is not justified, even when the measured affinities are comparable.

## Peptide-MHCII Thermodynamics and DM Function

DM function has been object of intense research in the past two decades, and we have recently gained important, though not conclusive, insights into its peptide editing activity. Other articles in this issue will review structure and editing function of DM, and only few aspects of DM activity will be highlighted here, which can be related to a thermodynamic/structural model of epitope selection.

Understanding DM function requires addressing several questions: (1) what renders a petide-MHCII complex susceptible to DM? (2) What is the effect of DM on the complex? (3) How does DM action result in selective binding of high-affinity peptides? Recent reports from different groups indicate that DM binds a short-lived MHCII intermediate whose N-terminal region features a specific conformation (39–42). This interaction seems to result in a stable, long-lived DM-DR complex after full dissociation of the prebound peptide has occurred (41). Still unclear is how the DM-labile conformation is generated. Historically, specific sources of binding energy have been indicated as responsible for generating DM-labile complexes (43–48), but now it appears that weakening of both H-bonds and “anchor-pocket” interactions results in susceptibility to DM (49–53). This latter observation would be consistent with a distributed model of peptide binding, where every position, to a different extent, affects the overall capacity of the peptide to bind, and the conformational flexibility of the complex. How does the overall binding property of the peptide determine DM susceptibility of the complex? One intriguing explanation would consider the interplay between compensatory mechanism and cooperativity as responsible for determining the probability by which the complex assumes a DM-labile conformation. Indeed, a complex whose formation is correlated to a smaller entropic penalty is expected to feature greater conformational mobility as compared to an isoenergetic complex whose formation requires a larger enthalpic contribution. As a consequence, the frequency increases at which regions within the N-terminal side of those complexes are disengaged from peptide interaction and are more amenable to DM. One observation in support of this hypothesis is the evidence that peptide-loaded MHCII conformers can be differentially susceptible to DM-mediated peptide release, and that the generation of the labile isomer is a function of the affinity of the bound peptide for DR (54), as well as the respective fraction of entropic contribution to the binding energy (37). Thus, when looking at the peptide release activity of DM, the thermodynamic strategy adopted by the peptide to bind seems to play a crucial role.

DM function needs to be considered in the context of peptide exchange, not only peptide release. The addition of an exchange peptide to the reaction is a confounding factor to the understanding of DM mechanism. Indeed it has been shown that exchange peptides are not simply replacing freshly dissociated peptides, but they intervene in, and possibly trigger the formation of a metastable intermediate that also includes the prebound peptide tethered to the MHCII (36). In particular, the role of the exchange peptide as “co-factor” in DM-mediated peptide exchange has been identified through its ability to convert DM-stable into DM-labile conformers (54).

For the active role played by the exchange peptide during a displacement reaction, the question of DM-mediated peptide exchange has been approached in terms of DM effect on the unfolding of the prebound peptide-MHCII complex and refolding around exchange peptide (36). When the analysis of cooperativity was performed in the presence of DM, no cooperativity could be observed in the release of the prebound peptide, contrasting what was observed for the intrinsic off-rate (35, 36). This evidence was interpreted as an indication that, once DM interacts with a complex, it promotes a dramatic disruption of the interactions between MHCII and the peptide, so that the typical coordinate unfolding of the intrinsic release cannot be measured. Interestingly, measuring cooperativity for the exchange peptide revealed that the latter needs to fold into the groove more efficiently than the prebound to displace it, and DM increases the energetic threshold that the exchange peptide has to overcome to displace the prebound peptide.

**Figure 1:**
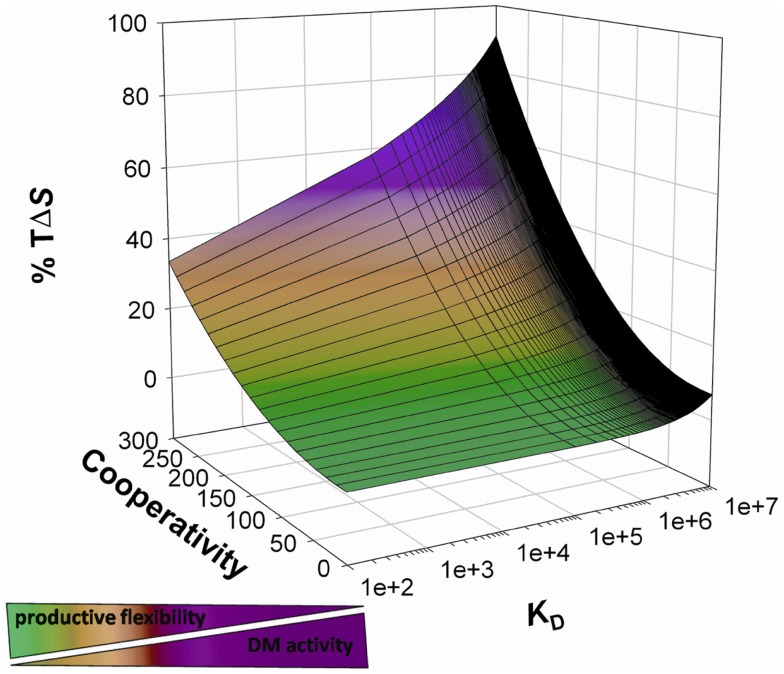
**Thermodynamic model of peptide binding and DM action**. The probability of a peptide binding to a given HLAII correlates with the probability of the interplay between cooperativity and compensation (here indicated as entropic contribution to free energy decrease) resulting in the optimization of all the available interactions. DM interacts with complexes featuring residual high entropy, promotes peptide release, and skews the presented peptide repertoire toward highly enthalpic complexes by increasing the effective free energy threshold for binding.

These results, along with structural (41) and molecular dynamic simulation analysis (55), supports a model in which DM interacts with complexes featuring a rearranged N-terminal and stabilizes the binding groove in a receptive state; DM shifts peptide exchange control from kinetic to thermodynamic. Moreover, DM interaction requires the engagement of MHCII residues, which would be otherwise elements of point system(s) with the peptide (39–42). Therefore, peptides are stably bound only when they effectively compete against DM to interact with DR residues for access to the N-terminal and if the overall binding energy is such to reduce the conformational lability. Such high-affinity, high-enthalpy binding would reverse the conformational rearrangement required for DM binding, thus leading to DM dissociation (Figure 1). Further studies are required to assess the correlation between thermodynamic signature of a peptide-MHCII complex, its conformational flexibility and susceptibility to DM-mediated peptide exchange. If confirmed, a similar mechanism would explain how the system reaches thermodynamic equilibrium with high-affinity binders in a timeframe compatible with epitope selection by the endosomal machinery.

## Conflict of Interest Statement

The author declares that the research was conducted in the absence of any commercial or financial relationships that could be construed as a potential conflict of interest.
